# Optimization of small molecule degraders and antagonists for targeting estrogen receptor based on breast cancer: current status and future

**DOI:** 10.3389/fphar.2023.1225951

**Published:** 2023-09-21

**Authors:** Jiaqi Yao, Yiran Tao, Zelin Hu, Junjie Li, Ziyi Xue, Ya Zhang, Yi Lei

**Affiliations:** ^1^ General Practice Ward/International Medical Center, General Practice Medical Center, West China Hospital, Sichuan University, Chengdu, Sichuan, China; ^2^ College of Life Sciences, Sichuan University, Chengdu, China; ^3^ West China-California Research Center for Predictive Intervention Medicine, West China Hospital, Sichuan University, Chengdu, Sichuan, China; ^4^ Precision Medicine Key Laboratory of Sichuan Province and Precision Medicine Research Center, West China Hospital, Sichuan University, Chengdu, Sichuan, China; ^5^ Department of Statistics, College of Liberal Arts and Sciences, University of Illinois Urbana-Champaign, Champaign, IL, United States

**Keywords:** estrogen receptor, selective estrogen receptor degrader, selective estrogen receptor covalent antagonist, structural optimization, small molecule drugs

## Abstract

The estrogen receptor (ER) is a classical receptor protein that plays a crucial role in mediating multiple signaling pathways in various target organs. It has been shown that ER-targeting therapies inhibit breast cancer cell proliferation, enhance neuronal protection, and promote osteoclast formation. Several drugs have been designed to specifically target ER in ER-positive (ER+) breast cancer, including selective estrogen receptor modulators (SERM) such as Tamoxifen. However, the emergence of drug resistance in ER+ breast cancer and the potential side effects on the endometrium which has high ER expression has posed significant challenges in clinical practice. Recently, novel ER-targeted drugs, namely, selective estrogen receptor degrader (SERD) and selective estrogen receptor covalent antagonist (SERCA) have shown promise in addressing these concerns. This paper provides a comprehensive review of the structural functions of ER and highlights recent advancements in SERD and SERCA-related small molecule drugs, especially focusing on their structural optimization strategies and future optimization directions. Additionally, the therapeutic potential and challenges of novel SERDs and SERCAs in breast cancer and other ER-related diseases have been discussed.

## Graphical Abstract

**Figure:**
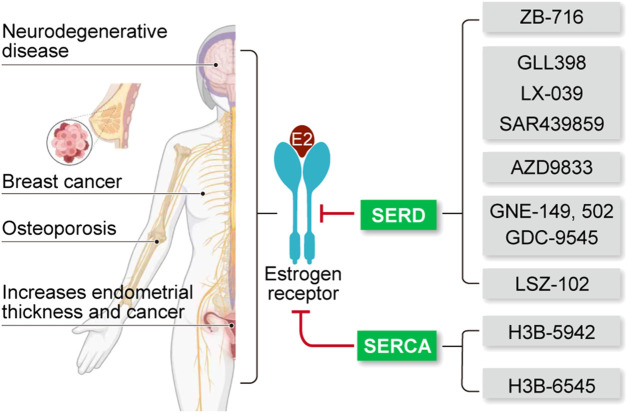
Small molecule drugs targeting ER for ER-related disease.

## 1 Introduction

Breast cancer is currently the most prevalent malignancy in women worldwide, and despite advancements in treatment, the death rate associated with it remains high (Siegel et al., 2020). Breast cancer is a heterogeneous disease and its heterogeneity can be manifested in molecular features, histological types, and therapeutic outcomes (Szymiczek et al., 2021). The status of estrogen receptor (ER), progesterone receptor (PR), human epidermal growth factor receptor 2 (HER2), and the proliferation marker Ki67 are significant predictive factors in breast cancer, playing crucial roles in the clinical decision-making process (Harbeck et al., 2019; Zhang et al., 2021a). With the development of gene sequencing technology, the classification of breast cancer that based on gene expression profiling (intrinsic subtyping) has emerged (Jamshidi et al., 2018; Szymiczek et al., 2021). Nowadays, the most widely accepted breast cancer intrinsic subtypes are Luminal A, Luminal B, HER2-Enriched, and basal-like. Luminal types A and B are characterized by positive expression of ER and/or PR. HER2-Enriched subtype is defined by overexpression of HER2 oncogene and low-to-absent ER expression. Basal-like subtype is characterized by the lack of expression of ER/PR and HER2 (Harbeck et al., 2019).

Approximately 80% of all breast cancers are ER+ (Siegel et al., 2020). The activation of ER by estrogen is a significant contributor to the development of ER+ breast cancer. This activation leads to various cellular responses that contribute to tumor growth and progression. Endocrine therapy, which targets the ER signaling pathway, has been a therapeutic treatment for this specific type of breast cancer, leading to potential mortality reduction of up to 40% (Herzog and Fuqua, 2022).

SERDs and SERCA-related small molecule drugs have emerged as the new types of endocrine therapy and have been developed rapidly in recent years. For example, Fulvestrant, a non-steroidal SERD, was approved in by the FDA 2007 for use as a monotherapy in postmenopausal women with luminal breast cancer (Soleja et al., 2019). Additionally, SERDs can be co-administered with other drugs, such as cyclin-dependent kinase 4/6 (CDK4/6) inhibitors (Liang et al., 2021). For instance, the combination of Palbociclib (an oral CDK4/6 inhibitor) and GDC-9545 (an oral SERD) demonstrated significant efficacy in a mouse tumor model, resulting in up to a 24% reduction in tumor size or degradation (Liang et al., 2021). There has been a significant increase in research focused on SERDs in recent years and several SERDs have entered clinical trials, as summarized in Table 1 (Wang and Tang, 2022).

**TABLE 1:** Clinical drug candidates of novel SERDs and SERCA.

Drugs	Sponsor	Phase	Indication	Intervention/Combination	NCT identifier	Status
ZB-716	EnhancedBio USA Inc.	II	Breast Cancer	ZB716 Palbociclib	NCT04669587	Recruiting
SAR439859 Amcenestrant	Sanofi	I	Breast Cancer	SAR439859 [14C]-SAR439859 micro tracer [14C]-SAR439859	NCT04940026	Completed
		I	Breast Cancer	SAR439859	NCT03816839	Active, not recruiting
		III	Breast Cancer	Amcenestrant Tamoxifen Amcenestrant-matching placebo Tamoxifen-matching placebo	NCT05128773	Terminated
		I/II	Breast Cancer	Amcenestrant Palbociclib Alpelisib Everolimus Abemaciclib	NCT03284957	Active, not recruiting
		II	Breast Cancer Metastatic	Amcenestrant Fulvestrant Anastrozole Letrozole Exemestane Tamoxifen	NCT04059484	Active, not recruiting
LX-039	ShandongLuoxin Pharmaceutical Group Stock Co., Ltd.	I	Advanced Breast Cancer	LX-039 tablets	NCT04097756	Enrolling by invitation
AZD9833 Camizestrant	AstraZeneca	III	Breast Cancer, Early Breast Cancer	Camizestrant Tamoxifen Anastrozole Letrozole Exemestane	NCT05774951	Not yet recruiting
		I	ER + HER2- Advanced Breast Cancer	AZD9833 AZD9833 with palbociclib AZD9833 with everolimus AZD9833 with abemaciclib AZD9833 with capivasertib AZD9833 with ribociclib AZD9833 with anastrozole	NCT03616587	Recruiting
		I	Healthy Subjects	Camizestrant Itraconazole	NCT05551897	Completed
		III	ER+, HER2-Breast Cancer	AZD9833 AZD9833 placebo Anastrozole Anastrozole placebo Letrozole Letrozole placebo Palbociclib Luteinizing hormone-releasing hormone (LHRH) agonist	NCT04964934	Recruiting
		I	ER+, HER2-, Metastatic Breast Cancer	AZD9833 AZD9833 with palbociclib AZD9833 with everolimus	NCT04818632	Recruiting
GDC-9545 Giredestrant	Hoffmann-La Roche	III	Locally Advanced or Metastatic Breast Cancer	Phesgo Giredestrant Docetaxel Paclitaxel LHRH Agonist Optional Endocrine Therapy of Investigator’s Choice	NCT05296798	Recruiting
		II	Endometrial Cancer	Giredestrant	NCT05634499	Recruiting
	Genentech, Inc.	III	ER+, HER2-, Locally Advanced or Metastatic Breast Cancer	Giredestrant Exemestane Fulvestrant Tamoxifen Everolimus LHRH Agonist Dexamethasone Mouth Rinse	NCT05306340	ER-Positive, HER2-negative, Locally Advanced or Metastatic Breast Cancer
		I	Breast Cancer	Giredestrant Procedure: Surgery	NCT03916744	Completed
		I	Breast Cancer	GDC-9545 Palbociclib LHRH Agonist	NCT03332797	Active, not recruiting
LSZ 102	Novartis Pharmaceuticals	I	Advanced or Metastatic ER + Breast Cancer	LSZ102 LEE011 BYL719	NCT02734615	Terminated
H3B-6545	Eisai Inc.	I	Estrogen Genes, Erbb-2 Breast Neoplasms	Palbociclib H3B-6545	NCT04288089	Active, not recruiting
		I/II	ER + Breast Cancer, ER + Tumor	H3B-6545	NCT03250676	Active, not recruiting
	Eisai Co., Ltd.	I	Breast Neoplasms	H3B-6545 Antihistamine	NCT04568902	Active, not recruiting

As research on SERD has progressed, several issues have emerged. For example, the use of acrylic groups in early SERD has been associated with adverse effects on the uterus (Xiong et al., 2017). In addition, the effectiveness of SERDs is significantly diminished when patients develop mutations in the *ESR1* gene, which encodes ERα (Baker, 2011; Dustin et al., 2019; Brett et al., 2021). Therefore, extensive research efforts are currently dedicated to developing safer and more potent SERDs that offer broader clinical benefits in the long term. Scientists have proposed the concept of SERCA to address drug resistance. SERCA targets the hot spot, a mutated amino acid residue known as C530, in a covalent manner (Puyang et al., 2018). The SERCA drug named H3B-6545 is currently in phase II of clinical trials (NCT03250676) as shown in Table 1 (Furman et al., 2022).

This review provides a comprehensive overview of the optimized pathways for SERD and newly synthesized derivatives, while also exploring their ongoing development status. We also propose emerging solutions such as SERCA to address concerns like drug resistance. Furthermore, the current development status and optimization strategies for both SERDs and SERCAs are described, aiming to provide innovative directions for their enhancement and potential applications in the treatment of breast cancer and other ER-related diseases.

## 2 Structure and function of ER

### 2.1 Molecular structure of ER

**FIGURE 1:**
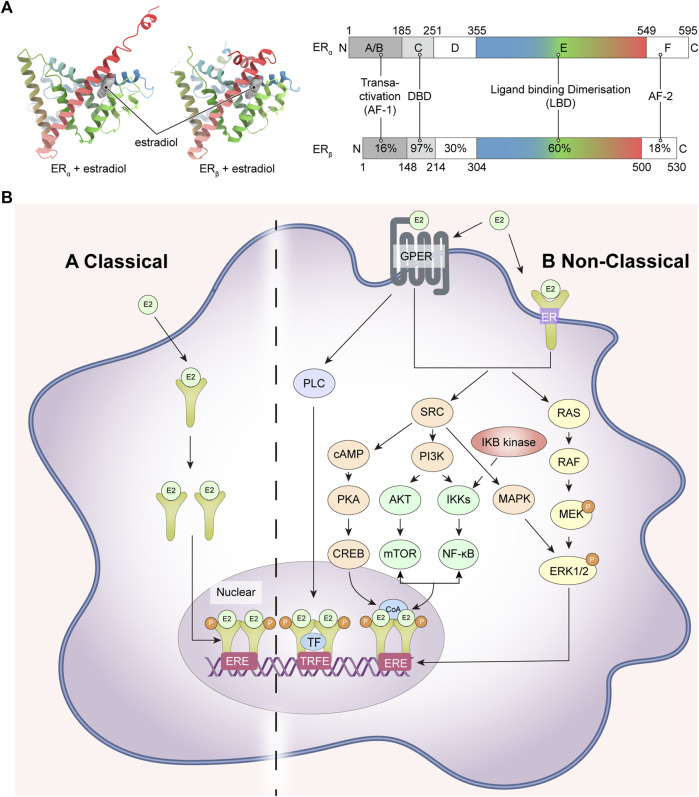
ER structures and ER-related signaling pathways. **(A)** ER crystal structure, ERα (PDB code: 1A52), ERβ (PDB code: 3OLS) on the left and schematic representations of the structure of ERα and ERβ on the right; **(B)** ER-related signaling pathways. ER, estrogen receptor; GPER, human epidermal growth factor receptor; RAS, Ras small GTPase; RAF, Raf kinase; MEK, mitogen-activated protein kinase; ERK1/2, extracellular signal-regulated kinase 1/2; SRC, non-receptor tyrosine kinase; PI3K, phosphatidylinositol-3 kinase; IKKs, IkB kinases; NF-κB, nuclear factor k-light chain enhancer of activated B cells; AKT, AKT murine thymoma virus oncogene; mTOR, mammalian target of rapamycin; CoA, coactivator; cAMP, cyclic adenosine monophosphate; PKA, protein kinase A; CREB, cAMP responsive element binding protein; MAPK, mitogen-activated protein kinase; PLC, activated phosphodiesterase C.

The classical ER is a member of the nuclear receptor superfamily and is composed of six modular domains, namely, A to F. It adheres to the conserved structure of the nuclear receptor family, including the DNA binding domain (DBD; C domain), the ligand binding domain (LBD; E domain), the hinge structural domain (D domain) and two transcriptional activation function domains, including AF-1 (in the A/B domain) and AF-2 (in the F domain) (Figure 1A) (Baker, 2011).

The DBD is the structural basis of the ER’s function as a transcriptional regulator of the nuclear receptor. Its primary function is to recognize and bind to specific DNA sequences known as estrogen response elements (EREs) that are located on the genome. The DBD is composed of two zinc finger structures, each containing four cysteine amino acids. These zinc finger motifs contribute to the structural stability of the DBD and enable it to interact specifically with the EREs (Deegan et al., 2011). The first zinc finger structure (ZF-I) recognizes a specific nucleotide sequence (5′-AGGTCA-3′) within the large groove at each end of the ERE double strand, and the second zinc finger (ZF-II) is responsible for homodimerization of the DBD. The LBD is a globular structural domain which encompasses several significant features, including a receptor binding site, a dimerization interface, and a coactivator and corepressor interaction function. The LBD is structurally composed of 11 alpha helices folded antiparallel, of which H12 plays an important role in binding ligands. H12 is not in the parallel region but acts as a “lid” by flipping outwards to make way for estradiol (E2) as it penetrates deeper into the internal hydrophobic pocket (Figure 1A). Due to its dynamic properties, H12 can trigger ligand-induced rearrangements within the LBD and plays a crucial role in the formation of both ER agonist and antagonist conformation. Additionally, H12 directly participates in the activation of AF-2 (Danielian et al., 1992).

The AF-2 structural domain encompasses the C-terminus of the ER, which is involved in the regulation of trans-activation function and LBD dimerization of ERα in conjunction with 4-hydroxy-tamoxifen (Koide et al., 2007; Arao and Korach, 2018). By contrast to AF-2, the AF-1 structural domain is linked to the N-terminal end, and when a ligand is present, the LBD undergoes a conformational change to dimerize and release the N-terminal structural domain, leading to AF-1 activation (The primary cause of ligand trans-activation following a conformational change of the LBD is thought to be H12) (Pike et al., 2000).

Estrogen-mediated signaling in mammals is regulated by a combination of two receptors: ERα and ERβ, encoded by the *ESR-1* and *ESR-2* genes expressed on human chromosomes 6 and 14, respectively (Greene et al., 1986). The E2-dependent activation of both can produce overlapping and unique physiological activities, and ERα also has a non-hormone-dependent activation that can be induced by a variety of cytokines, such as insulin-like growth factor (IGF) (Oesterreich et al., 2001). Although both ERα and ERβ follow the structural pattern of nuclear receptors and the DBD and LBD structural domains are highly conserved, the trans-activating functional regions are less homologous and it is generally accepted that the two receptors have different transcriptional activation functions. ERβ is metamorphosed upon binding to estrogen to form a homodimer that binds to the ERE to initiate gene transcription; it can also form a heterodimer with ERα that subsequently binds to the ERE (Cowley et al., 1997). In addition to the classical nuclear receptor-type ERs, there are other types of estrogen receptors including the G protein-coupled estrogen receptor (GPER), ER-X and Gaq-ER (Toran-Allerand et al., 2002; Micevych and Kelly, 2012; Vail and Roepke, 2019).

### 2.2 Physiological functions of ER

ER is widely distributed in various tissues throughout the body, including the breast, uterus, ovaries, bone, heart, and so on (Eyster, 2016). The expression of ER is influenced by various factors, including age, disease, and physical conditions. For instance, it has been shown that the increased stress load on the heart would upregulate ERα and ERβ expression in cardiac tissues (Bièche et al., 2001; Knowlton and Lee, 2012). ER is in multiple sites within the cell, including the nucleus, cytoplasm, and cell membrane. Once estrogen or other ligands bind to ER, the activated receptor translocates into the nucleus, binds to specific regions of EREs, and modulates the transcription of target genes. This process ultimately influences various cellular processes, including growth, development, and metabolism. This effect is known as the nuclear-initiated steroid signaling (NISS). However, the non-classical membrane pathway, known as membrane-initiated steroid signaling (MISS), in which estrogen acts as a signal transducer via novel G protein-coupled receptors (GPCRs) on the plasma membrane. This rapidly activates and influences regulatory cascades like mitogen-activated protein kinase (MAPK), phosphatidylinositol-3 kinase (PI3K), and tyrosine cascade (Figure 1B).

## 3 ER in multiple diseases

### 3.1 Cancer

#### 3.1.1 Breast cancer

According to the latest global cancer burden data, breast cancer accounted for 2.26 million cases in 2020 and remains the leading cause of cancer-related deaths among women globally (Wilkinson and Gathani, 2022). Extensive experimental data from both clinical and preclinical studies have consistently demonstrated the crucial role of estrogen and its receptors in the proliferation of breast cancer.

Single nucleotide polymorphisms (SNPs) in the ERα gene, also known as *ESR1*, have been implicated as significant factors associated with variations in the risk of developing breast cancer. Two common SNPs in the *ESR1* gene, known as PvuII and XbaI restriction site polymorphisms, have been studied extensively. The presence or absence of these restriction sites can influence the activity and expression of the ERα protein (Maguire et al., 2005). Research has also shown that growth factors and estrogen promote tumor cell proliferation by regulating the activation of the Ras-Raf-MEK-MAPK phosphate cascade or the PI3K/protein kinase B (AKT)/mammalian target of rapamycin (mTOR) pathway, which phosphorylates the ER in the nucleus. Additionally, they promote nuclear factor k light chain enhancer of activated B cells (NF-κB) signaling using growth factors, which drives breast cancer metastasis (Zhang et al., 2022). Meanwhile, the regulatory role of ER in breast cancer also involves co-regulatory factors, such as members of the non-receptor tyrosine kinase family. However, the regulation of ER in breast cancer is not fully understood. Further research is needed to investigate ER-regulated pathways and find solutions to address the issue of drug resistance in breast cancer.

Both SERDs and aromatase inhibitors are endocrine therapy drugs and have been utilized in breast cancer treatment (Second Oral SERD, 2023). Fulvestrant, the first approved SERD, has exhibited good antiproliferative efficacy. However, it does have limitations such as low aqueous solubility and the inability to be taken orally (Nardone et al., 2015). In a notable development, Elacestrant (RAD-1901), an oral SERD, received FDA approval in 2023 for the treatment of breast cancer (Bhatia and Thareja, 2023). Additionally, a randomized phase II trial of AZD-9833, a next-generation oral SERD and pure ERα antagonist, demonstrated a significant improvement in progression-free survival (PFS) compared to Fulvestrant in post-menopausal patients with ER + breast cancer who had previously received endocrine therapy (Turner et al., 2023).

#### 3.1.2 Endometrial cancer

Endometrial cancer is a common uterine disorder that is primarily caused by abnormal expression of ER (Yu et al., 2022). It is more prevalent in women with high estrogen levels who have ceased ovulating after menopause. In endometrial cancer patients, ERα expression levels in the uterine epithelium are generally higher than normal, even though ERα expression is generally downregulated during the secretory phase in the normal reproductive cycle (Bircan et al., 2005; Hu et al., 2005). GPER, which is highly expressed in aberrant endometrial cancer cells, has a similar expression trend to that of ERα, indicating that estrogen may activate intracellular GPER regulation via an intracellular pathway, which further increases the incidence of abnormal cell proliferation (Bulun et al., 2004).

Endocrine therapy, including the use of Fulvestrant, has been used more than 60 years in clinic for women with ER + endometrial cancer (Battista and Schmidt, 2016; Bogliolo et al., 2017). However, the low oral bioavailability of Fulvestrant has led to only mild activity in endometrial cancer treatment (Bogliolo et al., 2017).

### 3.2 Osteoporosis

Estrogen plays a crucial role in maintaining bone density (Khosla et al., 2012; Almeida et al., 2017). The decline in estrogen level can accelerate bone loss and increase the risk of developing osteoporosis. This commonly occurs in postmenopausal women and patients who have undergone surgical removal of their ovaries (E et al., 2016; Qi et al., 2023).

ERα in human osteoblasts can exert osteogenic effects through the CSE/H2S signaling pathway and modulation of NF-κB activity (Lambertini et al., 2017), and suppress bone cell apoptosis. The presence of ERα expression in osteoblast precursor cells, and the decrease in ERα expression during bone resorption and osteoblast maturation, suggest that the regulation and expression of ERα may also play an important role in osteoblast formation (Oreffo et al., 1999). Besides traditional genomic metabolic pathways, estrogen has also been reported to control osteoblast proliferation, differentiation, and apoptosis through activating the Ras/MAPK/extracellular regulated kinase, and PI3K/AKT signaling pathway via non-genomic pathway.

A recent study provided an overview of the efficacy and safety of the third-generation SERM, bazedoxifene, for the treatment of postmenopausal osteoporosis. Long-term treatment with bazedoxifene, up to 7 years, had demonstrated modest but significant improvements in bone mineral density in the lumbar spine and is generally safe and well tolerated (Yavropoulou et al., 2019).

### 3.3 Neurodegenerative diseases

Experimental studies have demonstrated that the levels of ERα and ERβ in hippocampal CA1 neuronal synapses in female rats decrease with age (Adams et al., 2002; Tao et al., 2021). These findings, along with significant gender differences in the pathophysiology of neurodegenerative diseases, highlight the important role of ER in conditions like Alzheimer’s disease (AD) and Parkinson’s disease (PD).

Several studies suggest that estrogen and its receptors have a modulating or neuroprotective effect on the brain (Shughrue et al., 2000; DeGregorio et al., 2014; Akwa, 2020). Jenna et al. and Yu’s team both found that after hormone therapy with E2, pathological deterioration was blocked, and Aβ accumulation was reduced in the hippocampus. Non-nuclear ERs such as GPER can activate extracellular signal-regulated kinase (ERK) and PI3K/AKT signaling pathways to improve memory impairment or prevent Aβ toxicity in experimental models of AD (Carroll and Pike, 2008; Kubota et al., 2016; Lai et al., 2019).

PD is primarily characterized by neurodegenerative motor dysfunction. Recent research has shown that selective activation of ERα can prevent the loss of dopamine transporter proteins in the striatum, and provide neuroprotective effects against nigral dopaminergic (DA) neurons in animal models of PD (D'Astous et al., 2004; Baraka et al., 2011). Estrogen has also been reported to have a protective effect against toxicity caused by 6-hydroxydopamine and the neurotoxin methylphenyltetrahydropyridine (MPTP) in DA neurons (Callier et al., 2002; Yoest et al., 2019). Hence, the modulation of E2 signaling using SERMs and drug analogs holds therapeutic potential for protecting PD-related nerves.

## 4 SERDs

Tamoxifen (**1**) (IC_50_ = 4.4 μM, EC50 = 1.41 μM) and Raloxifene (**2**) are commonly used SERMs for the treatment of breast cancer (Jordan, 2000). Modifications to the structure of these molecules, particularly the derivates with amino side chains, have led to the development of various compounds that induce specific conformational changes in the ER. These alterations affect the ER’s transcriptional activity and render it susceptible to degradation. Fulvestrant is an example of SERDs derived from these modifications. However, the clinical potential of Fulvestrant is limited by its poor exposure and the necessity for intramuscular administration (Dong et al., 2020).

**FIGURE 2:**
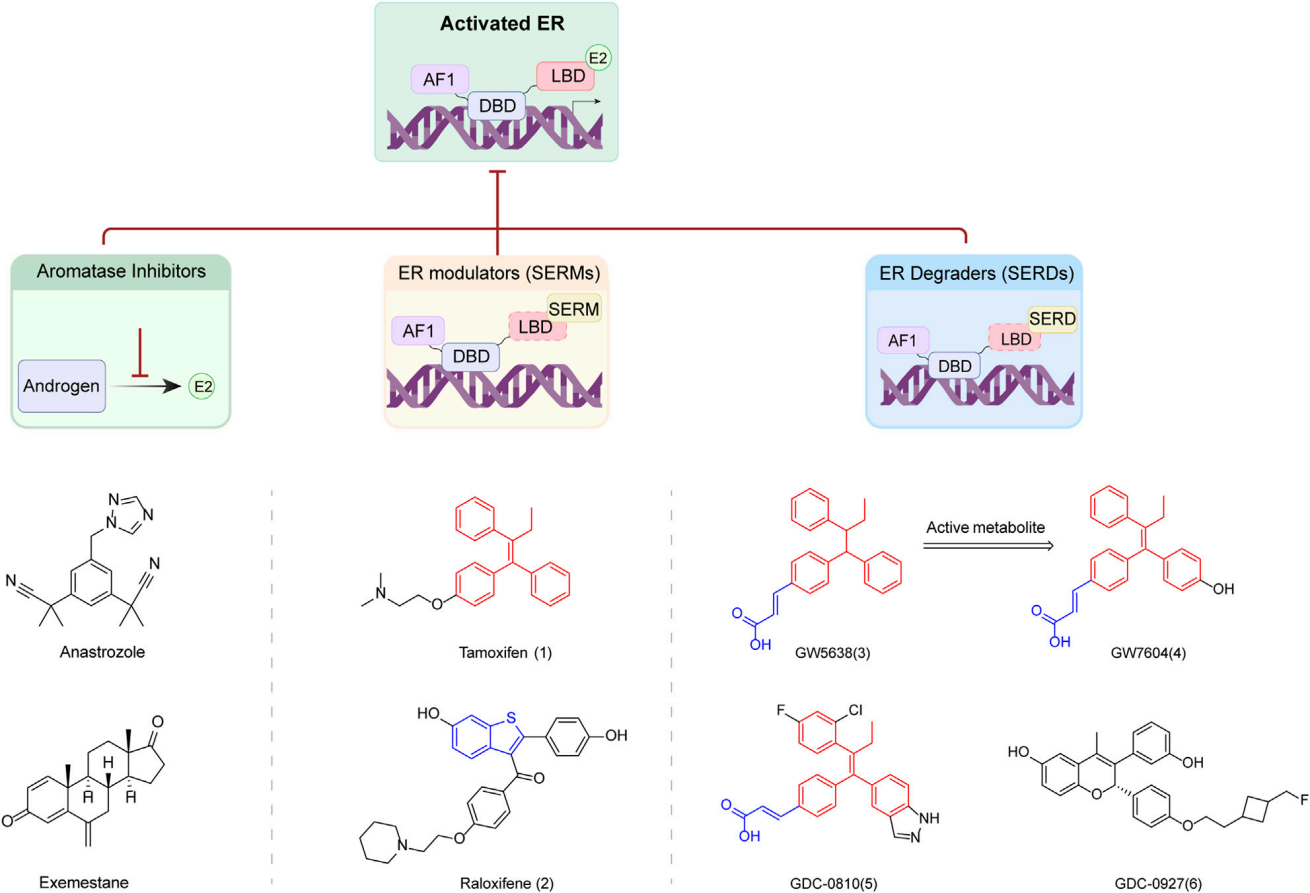
Mechanism of SERDs and related small molecule drugs.

To develop enhanced oral bioavailable ER degraders or antagonists, multiple studies have been conducted to screen new clinical candidates based on the structures of Tamoxifen, Raloxifene and Fulvestrant. These novel oral SERDs include GW5638 (**3**), GW7604 (**4**), GDC-0810 (**5**), GDC-0927 (**6**), and other compounds (Figure 2) (Willson et al., 1997; De Savi et al., 2015; Lai et al., 2015). This section focuses on the design of several recent oral SERDs based on the parent nucleus structure and side chain motifs of the above oral SERDs and according to the parent nucleus backbone. The pharmacological characteristics, and the advantages and disadvantages of these SERDs are summarized in Tables 2, 3.

**TABLE 2:** The pharmacological characteristics of novel SERDs.

Drugs	ER α binds IC_50_ (nM)	ER degradation EC_50_ (nM)	ER degradation in MCF-7 IC50 (nM)	Degradation efficiency/F%	PK parameters									Bioavailability%			Clearance/CL (mL/min/kg) iv	Volume of distribution (L/kg)/V_dss_	Lipophilicity log D7.4
					t_1/2_ (h)			C_max_ (ng/mL)			AUC (μg·h/mL)								
					Rat	Dog	Cyno	Rat	Dog	Cyno	Rat	Dog	Cyno	Rat	Dog	Human/cyno			
ZB-716	4.1	—	3.2	—	—	—	23.5	—	—	169.8	—	—	2.547	—	—	—	—	—	—
GLL398	0.21	—	1,800	—	—	—	3.9	—	—	3.51	—	—	36.9	15	—	—	—	—	—
LX-039	0.99	2.29	3.76	94	—	—	—	—	—	1,873	—	—	15.33	—	—	60.1	1.4	0.5	—
SAR439859	1.8	0.2	—	98	4.13	9.8	—	—	—	—	—	—	—	76	54	—	0.19	0.83	—
AZD9833	(pIC_50_ = 9.5)	—	—	—	1.2	6.1	—	—	—	—	0.83	3.2	—	31	45	—	23	—	2.6
D24	17.1	0.3	0.27	—	—	—	13.8 ± 1.5	—	—	2913.4 ± 904	—	—	9.98 ± 0.332	—	—	80.5 ± 4.2	1.67	1.4	—
GNE-149	0.66	—	0.053	100	—	—	—	—	—	—	—	—	—	31	8	13	19	—	4.8
GNE-502	0.5	—	—	93	—	13	—	—	—	—	—	—	1.8	—	—	—	7	7.7	2.2
GDC-9545	0.05	0.4	—	101	8	24	7.3	—	—	—	—	—	—	—	—	41	21	15	—
LSZ 102	0.2	—	—	83	14	4.1	—	240	268	—	1.849	7.713	—	33	12	—	19	0.9	—
Compound 40	3.2	1.1	1.0	94	4.6	—	—	764	—	—	4.629	—	—	—	—	67	7	2.7	—

**TABLE 3:** The advantages and disadvantages of novel SERDs and SERCA.

Drugs	Advantage	Disadvantage
ZB-716	Minimizes glucuronidation and sulfation, reduces first-pass metabolism *in vivo*, and improves bioavailability	Low level of drug exposure and clinical benefit to meet clinical phase III trial requirements
GLL398	Significant increases in drug exposure *in vivo*	—
LX-039	Low risk of dynamic drug interactions (DDI)	—
SAR439859	Demonstration of high anti-tumor activity in a mouse model of breast cancer xenografts	—
AZD9833	Low metabolic rate in human hepatocytes	Low drug clearance *in vivo* and *in vitro* in animal models
D24	High drug exposure, low clearance, and low oral toxicity during intravenous administration	—
GNE-149	High degradation efficiency *in vitro*	High lipophilicity, high binding affinity to plasma proteins, possible solubility and permeability problems
GNE-502	Lowest lipophilicity among reported ER ligands	—
GDC-9545	*In vitro* antiproliferative activity superior to other clinical candidates, potential efficacy in early-stage disease, antiproliferative effect superior to AI	Treatment-emergent adverse event in the trial
LSZ 102	—	Poor metabolic stability *in vivo*, lower ERα degradation activity
Compound 40	Fewer side effects of uterine augmentation	—
H3B-5942	Antagonistic potency against both ERα^WT^ and ERα^MUT^	The potency is critically dependent on covalency

### 4.1 ZB-716

**FIGURE 3:**
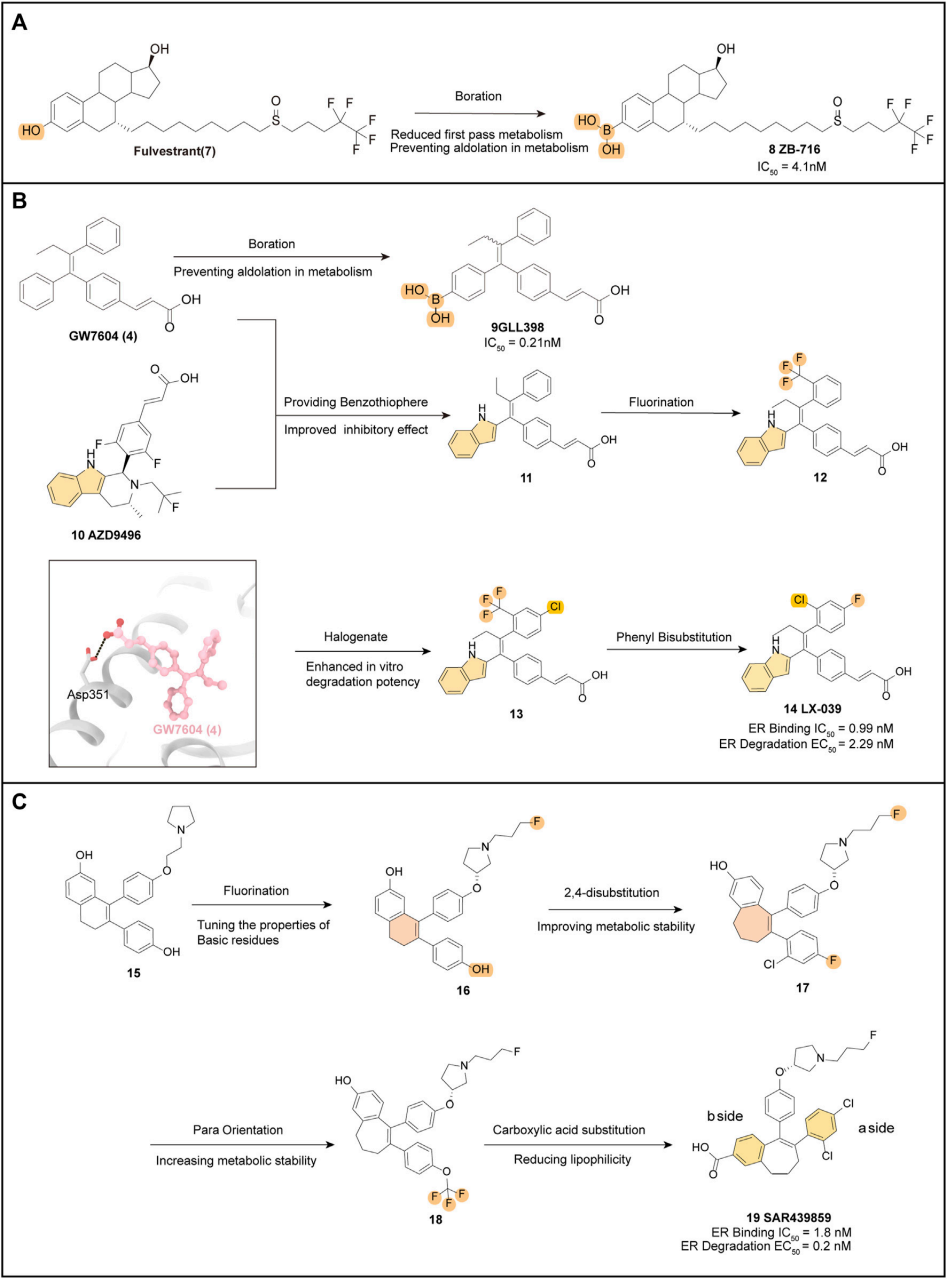
Optimization pathways related to derivatives with the estradiol backbone and triaryl ethylene backbone. Key optimized groups are marked in different colors. **(A)** optimization process of ZB-716; **(B)** several drug optimization processes based on the structure of GW7604 and the crystal structure of GW7604 (**4**) with ER is shown (PDB code: 1R5K); **(C)** optimization process of SAR439859.

ZB-716 (**8**) (IC_50_ = 4.1 nM) is an orally bioavailable SERD designed based on the fundamental parent nucleus structure of Fulvestrant (**7**) with a boronic acid moiety modification (Liu et al., 2016). When **7** is metabolized after oral administration, glucuronidation is a crucial metabolic pathway. However, by substituting the hydroxyl group at position 3 with a boronic acid group, aldolization of the drug molecule (**8**) can be effectively prevented (Figure 3A) (Liu et al., 2021). Molecular docking studies showed that ZB-716 (**8**) binds to ERα by the E2 portion of its structure, which has a long junctional chain of fluoropentylsulfonyl protruding through the open region formed by H10/11, H12, and H4/5. Due to the small size of the boronic acid moiety, its position in the binding capsule does not affect the localization of this fraction or the formation of hydrogen bonds between the binding fraction and the Glu353 and Arg394 sites. In pharmacokinetically relevant experiments, **8** also performed well, with high activity in a variety of tumor cells and Tamoxifen-resistant variants MCF-7/TamR (IC_50_ = 0.069 μM) and T47D/PKCα (IC_50_ = 0.037 μM). A pharmacokinetic study in mice demonstrated nearly 10-fold higher drug exposure and higher oral utilization of **8** compared to subcutaneous injection of **7**. And the linkage to plasma proteins protects **8** from oxidation, which may increase its half-life *in vivo*.

Therefore, **8** is a promising drug for the treatment of breast cancer patients with disease progression after prior endocrine therapy. Currently, **8** is in a clinical phase II trial (NCT04669587) and is expected to be administered in combination with palbociclib to improve the clinical benefit in ER+ patients.

### 4.2 GLL398

GLL398 (**9**) (IC_50_ = 0.21 nM), a SERD candidate, is designed based on the structure of GW7604 (**4**) and adopts a boronic acid functional group, mimicking the optimized model of **8**, to modify **4** and block its primary site of phase II metabolism (Figure 3B).

The eutectic structure (PDB: 3ERT) clearly shows that the boronic acid group of **9** is more tightly attached than the hydroxyl group of **4**, forming hydrogen bonds with residues Glu351 and Arg394 of the ERα protein. Furthermore, the boronic acid group in **9** forms a hydrogen bond with the carbonyl oxygen of the main chain of Leu387, resulting in increased stability when compared to **4** (Guo et al., 2020).

The binding affinity of **9** to ERα is almost 10-fold higher than that of Tamoxifen, and this has been supported by free energy experiments. The incorporation of boron modification helps enhance the pharmacokinetic profile of **9** and provides significantly higher drug exposure *in vivo* (AUC = 36.9 μg h/mL) (Liu et al., 2017). As an orally administered SERD, **9** exhibits high affinity and potent antitumor efficacy, which make it a promising candidate for the treatment of breast cancer. Furthermore, the successful application of the boronic acid structure for enhanced oral bioavailability in **9** also contributes to the development of other novel SERDs.

### 4.3 LX-039

The eutectic structure of **4** (Figure 3B) (PDB: 1R5K) and molecular docking simulations of AZD-9545 (**10**) show that the indole structure of **10** forms a hydrogen bond with Leu352 of ER, anchoring the entire drug molecule in the LBD of ER. The phenol structure of **4** interacts strongly with the residues within the LBD pocket of ER. Therefore, compound **11** replaces the phenol structure of **4** with an indole group, which enables the NH of the indole to form a hydrogen bond with Leu346, while the remaining components of the molecule interact hydrophobically within the binding pocket, leading to excellent inhibition of ER (IC_50_ = 0.28 nM). SAR exploration based on **11** focused on the mono substitution of lipophilic groups at the pro-, inter-, and para-positions of the benzene ring, leading to the development of the fluoroalkane-substituted compound **12**. Further disubstitution revealed that the 2,4-disubstituted drug (**13**) had better *in vitro* degradation than **12** (Dong et al., 2020). Among several 2,4-disubstituted attempts, LX-039 (**14**) was ultimately selected (Figure 3B). Due to the enhanced hydrogen bonding interaction between the NH of the indole moiety and the Leu346 residue of ERα, which increases the binding affinity of the drug molecule to ER (Figure 3B), the mode of interaction of **14** (IC_50_ = 0.99 nM, EC_50_ = 2.29 nM) with the protein suggests that the tetra-substituted ethylene with a triple aryl substituent has a unique propeller conformation, with the three large aryl groups “compacted” into a relatively small space by covalent bonding with ethylene. This compaction of the three large aromatic groups into a small space allows a spatially restricted group rotation within the molecule, which provides a bioactive conformation favoring the degradation of ER (Kuramochi, 1996).

**14** exhibits potent degradation of ER in MCF-7 breast cancer cells (IC_50_ = 1.53 nM) and efficiently inhibits MCF-7 cell proliferation (IC_50_ = 2.56 nM) *in vitro* assays. It also demonstrates a favorable Pharmacokinetics (PK) profile across species (AUC = 15,329 nM•h, F = 60.1%). Notably, the greatest advantage of **14** is its high safety profile, which has a purer ER antagonistic effect. In a rat uterine growth inhibition assay, it achieves 94% inhibition with a clean CYP inhibition profile, indicating a low risk of dynamic drug interactions (DDI). Therefore, **14** has the potential to be used in combination with CDK and GPCR inhibitors to achieve better inhibition and also has high promise as an oral SERD agent for the treatment of ER+ breast cancer. Currently, human clinical trials are underway to determine its PK profile and efficacy (NCT04097756) (Lu et al., 2022a).

### 4.4 SAR439859 (amcenestrant)

Building upon the parent nucleus structure of early oral SERDs such as GW7604 (**4**) and AZD9496 (**10**), scientists initially obtained new starting compounds **15** after medium-throughput screening (MTS) of MCF-7 breast cancer cell lines using an intracellular protein immunofluorescence assay.

The optimization strategy focused on selecting the appropriate O-R side chain, and it was discovered that the substitution of fluoropropyl could improve the ERα degradation activity by modulating the nature and residual position of the basic residue. The fluoropropyl-pyrrolidine degradant side chain (**16**) was further explored in different scaffolds.

Introducing a haloaryl group in place of the **b-side** phenol at the hydroxyl position of the **a-side** benzene ring resulted in interference, however, compound **17** retained degradation potency while also improving metabolic stability. Optimization of the SAR of the **b-side** benzene ring led to a significant increase in degradation potency and good metabolic stability for compound **18**, obtained by para-substitution of OCF3, but with a high lipid-water partition coefficient (LogD) value and some pharmacokinetic defects. To reduce the LogD of the compound, the research team substituted the hydroxyl group on the **a-side** benzene ring with a carboxylic acid, and then re-substituted the **b-side** benzene ring with a halogen, resulting in **19**. This compound showed high degradation efficacy (98%) and good potency (EC_50_ = 0.2 nM), comparable to the *in vitro* activity of Fulvestrant, and exhibited significant metabolic stability (El-Ahmad et al., 2020).

SAR439859 (**19**) is a compound with a unique fluoropropyl pyrrolidinyl side chain. Unlike Tamoxifen, it did not exhibit agonist activity on the ERα receptor in the uterus. This suggests that SAR439859 induces full antagonist activity on ERα, thus reducing the risk of uterine carcinogenesis. *In vitro* selectivity assessments, it showed no off-target activity (IC50 < 1 μM), while *in vivo* pharmacokinetic profiles demonstrated low to moderate clearance (0.03–1.92 L h^−1^ kg^−1^), low to moderate volume of distribution (Vdss = 0.5–6.1 L/kg), and good cross-species bioavailability. Furthermore, SAR439859 has demonstrated high anti-tumor activity in breast cancer mice xenograft models without causing weight loss (Shomali et al., 2021). Based on these promising results from preclinical *in vivo* pharmacological assays and favorable pharmacokinetic properties, SAR439859 (**19**) has progressed to phase III clinical trials (NCT05128773).

### 4.5 AZD9833 (camizestrant)

**FIGURE 4:**
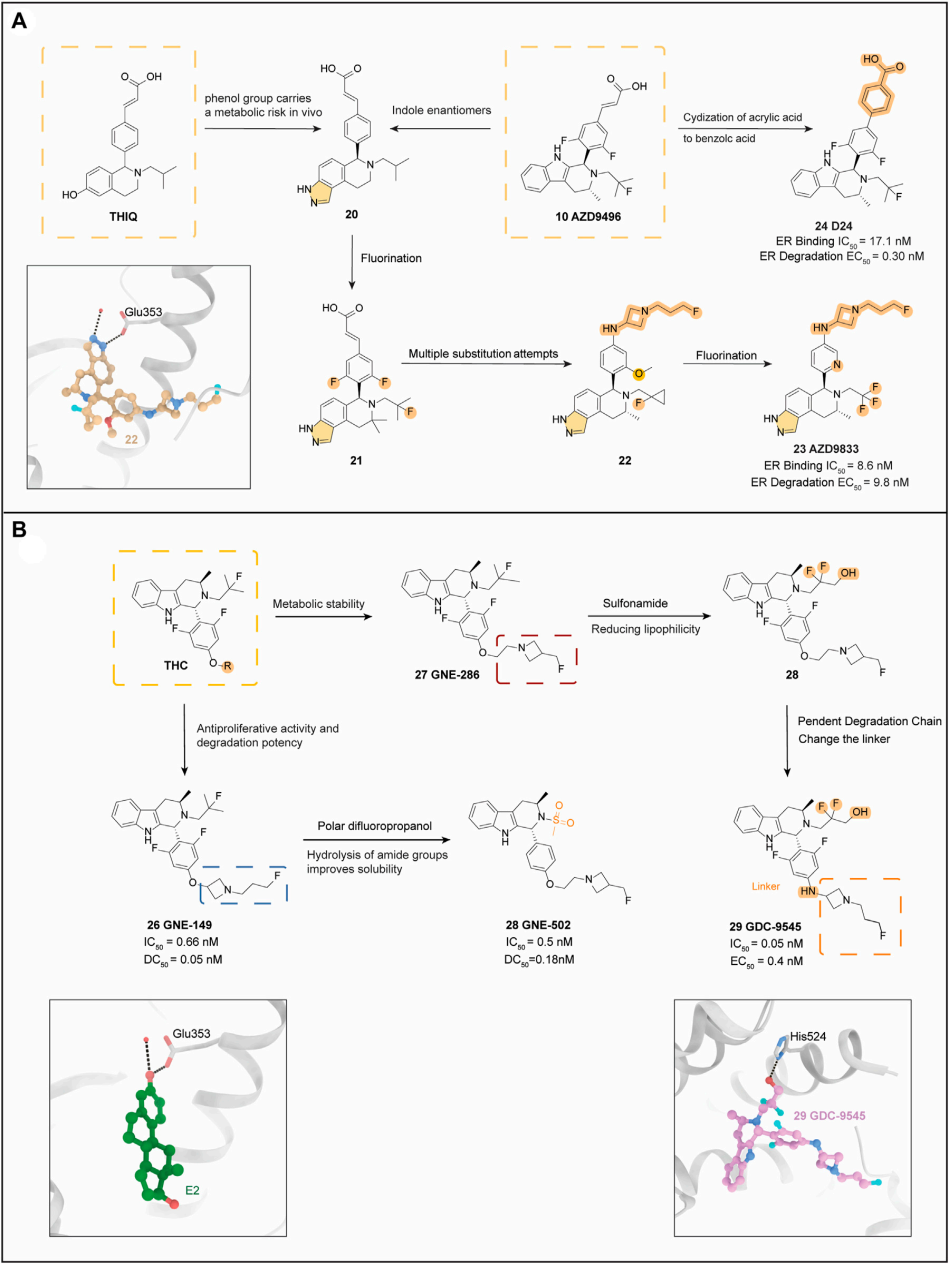
Tricyclic indazole backbone derivatives. **(A)** using THIQ as the core, optimization processes were conducted for both AZD9833 and D24. Both of these derivatives were obtained by referencing the structure of AZD9496. The crystal structure of ER ligand binding domain with compound **22** (PDB code: 6ZOQ); **(B)** using THC as the core, optimization processes of GNE-149, GNE-502, and GDC-9545. The crystal structure of the complex formed by estradiol (E2) and ER is shown in the bottom left side (PDB code: 5DXB). The crystal structure of ER with compound 29 is shown in the bottom right side (PDB code: 7MSA).

The tetrahydroisoquinoline scaffold, designed to mimic the structures of E2 and coumarin, showed ER degradant and antagonist characteristics. However, the inclusion of a phenol moiety in this scaffold resulted in metabolic risk *in vivo*. Therefore, the J.S. Scott team introduced the indole moiety to create a series of new compounds. Among these compounds, **20** (which incorporates an indole enantiomer) showed strong degradation potency but had slightly higher lipophilicity (ΔlogD7.4 + 0.4). Increasing the number of alkyl chain substitutions improved the potency of compound **21,** while fluorination of the aromatic ring enhanced its metabolism in hepatocytes. Protein blotting studies demonstrated that **21** was a potent ERα degradation agent in MCF-7 cells. Moreover, it exhibited significant tumor growth inhibition in a xenograft model and was well tolerated (Figure 4A) (Scott et al., 2016).

Through the incorporation of multiple alkyl chains and indole substituents, compound **22** was found to have higher degradation potency and lower lipophilicity compared to the other compounds tested. The X-ray crystallographic analysis of compound **22** (PDB: 6ZOQ) revealed that it binds to an ERα ligand-binding structural domain construct and that the tricyclic indazole core interacts with conserved water and Glu-353. However, this backbone differs from the previously reported tricyclic [6.5.6] indole core of **10** (De Savi et al., 2015), in that the tricyclic indazole lacks an indole NH hydrogen bond donor in the central ring that interacts with the backbone carbonyl group of Leu346, which is not conducive to optimization. Pharmacological experiments also revealed that **22** exhibited poor bioavailability (3%) in rats. To address this limitation, the team reduced the alkalinity of the piperidine ring nitrogen by aliphatic fluorination of the methoxyphenyl and pyridinyl side chains, resulting in reducing the volume of distribution, where the pyridinyl group is less lipophilic than methoxyphenyl. This result, which was compounded by the fact that the pyridinyl group is less lipophilic than the methoxyphenyl group, led to the development of compound **23**. After the addition of a third fluorine atom to the ethyl side chain, it met the team’s predetermined potency and lipophilicity criteria, while maintaining a low metabolic rate in human hepatocytes (Scott et al., 2019).

In terms of molecular conformation, AZD9833 (**23**) exhibits a binding pattern similar to that of **22**, with the tricyclic core of the molecule tightly wrapped by hydrophilic residues that form a contract similar to that seen previously. The basic group of **23** is also close to Val533 and Asp351, as is the acrylic group. The bioavailability of **23** is moderate in both mice (16%) and rats (19%), with good pharmacokinetic properties. Additionally, due to its high solubility, good permeability, partial absorption, and moderate clearance, it is anticipated that this compound will have good bioavailability in humans (F > 40%). **23** also exhibited low risk in an *in vitro* safety assay and antagonistic potency in ERα wild-type and Y537S ERα-expressing mutant MCF-7 cells. Currently, it is in phase III clinical trials (NCT04964934).

### 4.6 D24

D24 (**24**) is a newly developed derivative of AZD9496 (**10**) (Zhang et al., 2021b). The acrylic acid side chain of **10** had previously shown high side effects on the uterus, necessitating a series of optimizations focused on the hydrogen bonding and hydrophobic interactions of key amino acids in the proximity of H12 following a conformational analysis.

The team employed the Desmond module to analyze key amino acids at the binding site of ERα to SERD and utilized various methods to evaluate the contribution of individual amino acids to protein-ligand interactions. For instance, they evaluated the sites of intramolecular hydrogen bonding and hydrophobic interactions by counting alanine mutations. The multiple substitution results indicated that the 2,6-difluorophenyl scaffold is crucial for both ERα binding and degradation. Acrylates are commonly used in clinical SERDs due to the critical hydrogen bonding interactions that the acrylate side chain forms with Leu536 and Tyr537. However, acrylates have been known to cause uterine hyperplasia, leading to increased attention devoted to addressing this metabolic issue (Xiong et al., 2017). Changing the acrylate to an aliphatic acid was found to be an effective solution to this problem. Therefore, the researchers cyclized the acrylic acid into benzoic acid, producing compound **24**. This new compound demonstrated significantly greater degradation efficacy and better MCF-7 anti-proliferative activity than **10** (IC_50_ = 17.1 nM; EC_50_ = 0.30 nM). Analysis of the molecular docking of **24** revealed that the benzoic acid portion of the compound is believed to extend from the binding pocket between α-helix 3 (H3) and 11 (H11), resulting in increased binding affinity (Figure 4A) (Brzozowski et al., 1997). Although various heterocyclic substitutions were attempted to explore their effects, none of them increased the antitumor activity, further emphasizing the importance of the benzoic acid side chain in ERα degradation.

In the analysis of biological assessment, compound **24** was found to exhibit exceptional ERα binding affinity, ERα degradation efficacy, and anti-proliferative activity, surpassing compound **10**. Furthermore, it displayed superior pharmacokinetic performance, with good drug exposure and low clearance observed in intravenous administration. *In vitro* PK profiling also revealed greater metabolic stability of compound **24** compared to compound **3**, further validating the drug-forming properties of the benzoic acid motif. Importantly, preclinical toxicity studies showed that compound **24** exhibited low oral toxicity, indicating its potential for further clinical evaluation.

### 4.7 GNE-149, GNE-502

In the realm of second-generation oral SERDs, such as GDC-0810 (**5**) and GDC-0927 (**6**), clinical trials have demonstrated lower oral doses, but with a consequential increase in drug burden.

Additionally, the tetrahydro carboline has emerged as a promising novel backbone for the development of SERD, based on the structures of compound **6** and **10**. These compounds have shown excellent oral exposure and metabolic stability. Through experiments involving chlorine atom substitution, the side chain of this compound was carefully selected to enhance its antiproliferative activity and degradation potency. Additionally, the tail of the compound was substituted with a fluorine atom, further augmenting its properties.

A new azetidine ring was developed by opening the azetidine ring and cycling it onto the carbon near the oxygen atom, resulting in GNE-149 (**25**). This modification resulted in improved degradation efficiency, with IC_50_ = 0.66 nM, DC_50_ = 0.05 nM, and S_inf_ = 100% (Liang et al., 2020). In T47D cells, **25** was more efficiently degraded compared to **5** and was more metabolically stable *in vitro* compared to **6**. Moreover, optimization with **25** enabled the drug to achieve the same effect at a significantly reduced dose, which could potentially reduce the pill burden. In addition, experimental results in multiple cancer cell models demonstrated an excellent and complete *in vitro* and *in vivo* antagonist profile and antitumor activity in the mutant phenotype of **25**. Tetrahydrocarboline was also proved to be a promising SERD backbone, with good oral bioavailability (F% _Rat_ = 19.31%, F% _Dog_ = 8.49%, C_yno_ = 13.28%).

Despite the promising results of GNE-149 (**25**), the analysis of its crystal structure bound to ERα revealed some challenges in further optimization (Figure 4B) (PDB: 6ZOQ). Specifically, the formation of three hydrogen bonds within the binding site and the recruitment of the agonist conformation of H12 closed the site to the solvent, indicating high lipophilicity and potential solubility and permeability issues. Moreover, the binding affinity of **25** to plasma proteins was observed to be very high, which could potentially impact the drug’s efficacy.

However, through relevant pharmacodynamic experiments, Jason R. Zbieg based on crystal structure analysis (PDB: 5DXB), found that better coordination of key ionic interactions between azetidine and Asp351 substantially enhanced the degradation of the drug (Zbieg et al., 2021). Together with the analysis, which revealed that the hydrolysis of the amide group led to improved solubility, the final choice of the sulfonamide derivative as the side chain improved the degradability, while the removal of the fluorine atom from the central ring further reduced the lipophilicity yielding GNE-502 (**26**) with low lipophilicity and essentially unchanged degradation potency. The final selection of the sulfonamide derivative as the side chain improved the degradability of the drug, while the removal of the fluorine atom from the central ring further reduced the lipophilicity. This led to the development of **26** which showed decreasing lipophilicity and retained unchanged degradation potency.

Notably, **26** represents the least lipophilic of the reported ER ligands, and its sulfonamide derivative side chain showed a strong and potent antagonistic effect *in vitro* antagonist assays (IC_50_ = 0.5 nM, DC_50_ = 0.18 nM, S_inf_ = 93%). In addition, it demonstrated good *in vivo* efficacy in a mouse tumor xenograft model (AUC = 1.8uM*hr), indicating a significant advancement in the development of SERD.

### 4.8 GDC-9545 (giredestrant)

GNE-286 (**27**), which still relies on the THC parent nucleus structure, was developed by adding a basic side chain that optimized ER antagonism and degradability while ensuring good metabolic stability. Subsequently, a polar difluoropropyl alcohol was introduced into the pyridine moiety at various positions to reduce lipophilicity (**28**). To achieve greater degradation efficiency, a tetrameric azetidine was chosen as the pendent degradation side chain, with an amine junction instead of an oxygen junction. This led to the development of GDC-9545 (**29**) (IC_50_ = 0.05 nM, EC_50_ = 0.4 nM), which exhibited low lipophilicity, high solubility, and high permeability, making it an excellent candidate for oral degradation agents (Chen et al., 2022). Currently, **29** is commonly used in combination with CDK4/6 inhibitors in the treatment of HR-positive breast cancer (NCT03332797) (Hernando et al., 2021).

The eutectic structure of **29** with ERα (Figure 4B) (PDB: 7MSA) shows that the ionic interaction of the charged aziridinium nitrogen (pKa = 8.1) with the Asp351 side chain in H3, making **29** highly effective against the *ESR1* hotspot mutant Y537S. Additionally, the primary alcohol located in the fluorine-containing side chain forms strong hydrogen bonds with His524 of H11, resulting in a tighter binding of H11 to **29**. Finally, the interaction of the ligand with H3 is further strengthened by the weak hydrogen bonding from the THC core indole NH to the carbonyl oxygen of the Leu346 backbone in H3. All of these interactions contribute to the binding potency of the compound (Liang et al., 2021).

In clinical trials, **29** demonstrated the same blocking effect as GDC-0927 (**6**) with only 1% of the dosage of GDC-0927. Various trials have also demonstrated that **29** exhibits excellent *in vitro* anti-proliferative activity compared to other clinical candidates and approved drugs, and in combination with CDK4/6 inhibitors, it has excellent degradation efficacy *in vivo* (Shao, 2021).

### 4.9 LSZ 102 and its derivatives

LSZ 102 (**30**) is a compound with a benzothiophene backbone that was developed based on the optimization of Raloxifene (**2**) (Lu et al., 2022b). The introduction of ether-linked **31** at the junction between the benzothiophene backbone and the side chain significantly increased ERα antagonist activity compared to other compounds. In further optimization, the unsaturated bond of cinnamic acid in **a-side** chain of **31** was retained, aiming to improve the low bioavailability and high clearance of the compound. Proximal substitutes with potentially metabolically unstable phenolic-containing functions were also introduced to the **b-side** chain of **31**. The incorporation of difluoroethyl with the P-fluoro group found in earlier analogs yielded **30**, which exhibited an acceptable PK profile, 12% oral bioavailability, and the most potent experimental ERα antagonist and degradation agent. Docking analysis of **30** with ERα-LDB (301-553) demonstrated that it adequately occupied the available space inside the LBD.

In terms of pharmacodynamics, **30** performed well. A dose-dependent assessment of ERα degradation demonstrated that **30** was more effective compared to Fulvestrant (**7**) (IC_50_ = 0.2 nM vs. 1.2 nM). Moreover, at well-tolerated dose levels, **30** exhibited potent antitumor efficacy and PD marker inhibition in the MCF-7 human breast cancer model. Currently, **30** is in phase I/Ib trial to evaluate its efficacy in advanced or metastatic ERα+ breast cancer (Scott and Barlaam, 2022). Preliminary results show that **30** is well-tolerated and has a manageable safety profile (Artault et al., 2022). However, poor metabolic stability and low ERα degradation activity *in vivo* have led to unsatisfactory results in phase I trials.

Many phenol-containing SERDs have been limited in late-stage clinical trials due to metabolic issues and pharmacokinetic problems. To improve pharmacological characteristics and pharmacokinetic properties, optimization of a new compound **36**, was conducted (Lu et al., 2022b).

**FIGURE 5:**
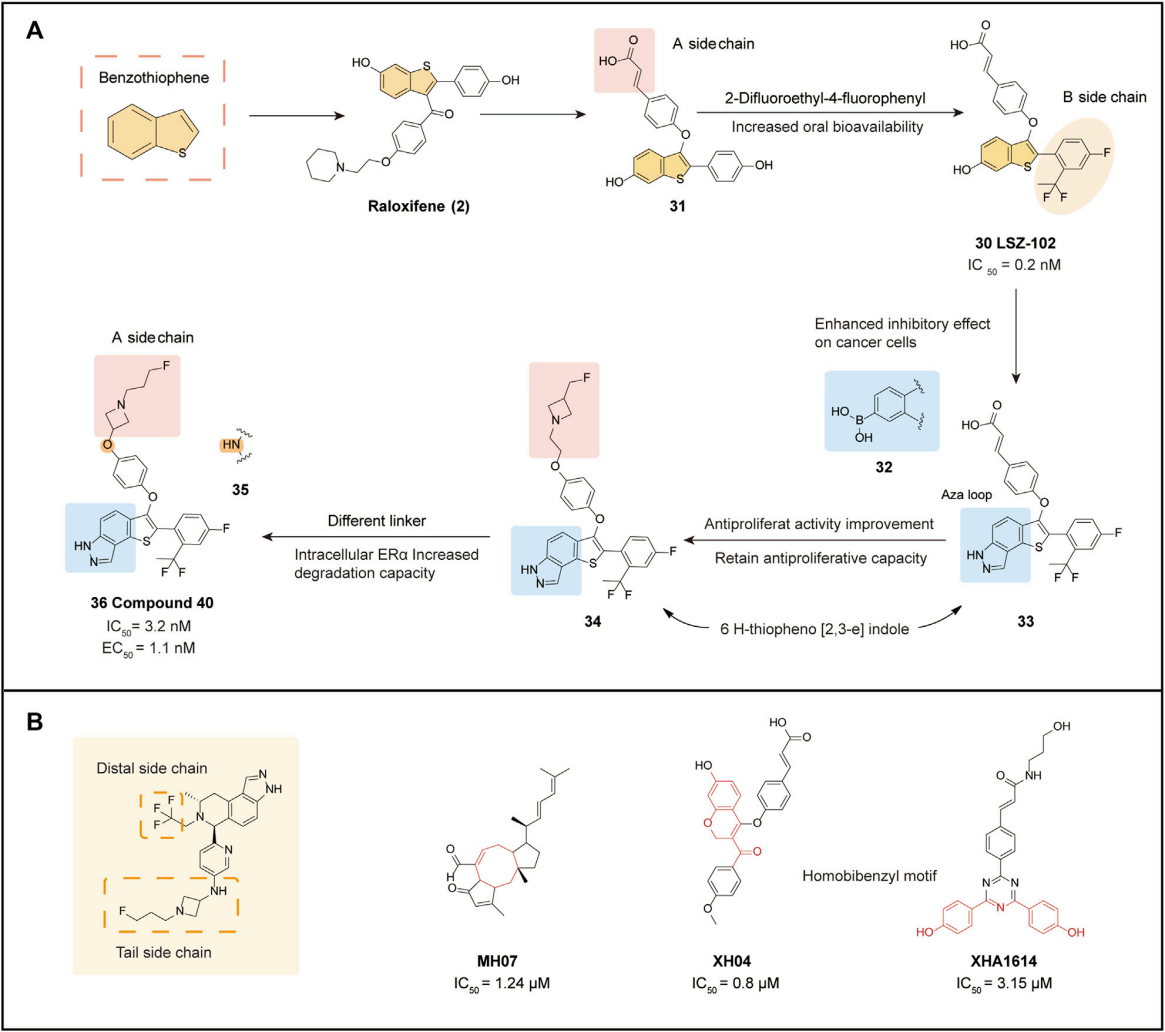
Benzothiophene derivatives and SERD optimization design. **(A)** optimization processes of LSZ-102 and Compound **40**; **(B)** SERD optimization strategies and some new core structures that may be applied to ER-targeted drugs.

Based on the structure of **30**, both the boronic acid compound **32** and the Thio indazole compound **33** were optimized to gain inhibition of cancer cells. Spatially, **33** has nearly the same molecular conformation as **30**, with the -NH- in the indazole ring overlapping with the phenol group in **30** and interacting with two key residues in the ERα LBD (Glu353, Arg394) through key hydrogen bonding interactions (Tria et al., 2018). In particular, two isolated hydrogen bonds to Glu353 and Arg394 were found in the **33** and ERα complexes, leading to a sharp reduction in the antiproliferative ability of the isomer 1H-thieno[3,2-f] indazole, which was due to the incorrect orientation of the nitrogen atom and interaction with Arg394. The contact to the fluorine atom and the His524 hydrogen bond suggests that the para-substitution pattern should be retained (Figure 5A).

In a study of **33** screened for Aza ring as well as carboxylic acid functional groups, it was found that under conditions where 6 H-theine[2,3-e] indazole was substituted with the Aza ring, the side chain underwent fluoroethyl substitution resulted in the development of a well-behaved **34** (IC_50_ = 1.1 nM), with a nearly 4-fold increase in antagonism (EC_50_ = 0.9 nM/86.7%) that caught up with **30** in ER degradation assays for the first time. Inspired by third-generation SERDs, the team introduced reverse azetidine and pyrrolidine in **34**. From this series of derivatives, **35** and **36** were obtained, with **36** showing comparable activity in MCF-7 growth inhibition compared to **34**, but with a significantly higher maximum efficacy of intracellular ERα degradation of more than 90% (EC_50_ = 1.1 nM/94%). There were no significant differences in terms of N-linkage (**35**) and O-linkage (**36**) affecting ERα function. However, moderate human ether-à-go-go-related gene (hERG) inhibition was observed in all N-linked compounds (including **35**), which may limit their long-term use, and therefore **36** with high hERG inhibition is a better choice. In efficacy trials, **36** demonstrated a significant improvement in oral bioavailability (67%) compared to **29**. Based on ERα protein blot analysis, **36** was a more potent ERα degrader than **29** in several ER+ breast cancer cell lines. Additionally, uterine wet weight and histological assessment showed **36** to be a complete antagonist, reducing the side effects of uterine hyperplasia. Molecular docking results demonstrated the ionic interactions formed between the nitrogen atom and Asp351 when the acrylic acid side chain was replaced with a reverse azetidine. This not only enhanced affinity for wild-type ERα but also disrupted the constitutively active conformation of the Y537S and D538G ERα mutants, which further conferred degradative and antagonistic activity on ERα mutants. Overall, **36** represents a potential and promising oral SERD for ER+ breast cancer treatment, and further evaluation is ongoing.

### 4.10 Future design directions for SERD structures

The optimization of oral SERDs typically focuses on three aspects: the distal side chain, the tail side chain, and the backbone, Various strategies can be employed to optimize each aspect (Figure 5B).

The Distal Side Chain:

1) The original SERDs used a phenolic hydroxyl group as a distal side chain, but this was later eliminated due to a low binding affinity to the receptor.
2) Fluorine substituents are commonly used as distal side chains, such as tri-substituted fluoroalkanes in LX-039 and AZD9833, difluoroethyl in LSZ-102, and 2-difluoroethyl-4-fluorophenyl in compound **40.** Fluorine substitution improves degradation potency, and polarity can also reduce lipophilicity, as seen in GDC-9545, which used difluoropropanol.
3) Substitution of the phenolic hydroxyl group with a boronic acid group is an emerging optimization approach, as seen in ZB-716 and GLL398. The smaller size of the boron atom results in a more stable complex due to hydrogen bonding, and the substitution of boronic acid groups blocks primary metabolism, increasing oral bioavailability.
4) Side chains of sulfonamide derivatives, exemplified by GNE-502, are more specific and can improve drug degradation, providing ideas for future drug optimization.

The Tail Side Chain:

1) The acrylic acid side chain (cinnamic acid), commonly used on the benzene ring, forms critical hydrogen bonding interactions with Leu536 and Tyr537 (PDB: 5ACC) (Wu et al., 2005), leading to conformational changes in the AF2 structural domain, and promotes ERα degradation. However, clinical trials have also shown that acrylic acid SERD has a partial agonistic effect on rat uterine tissue (Kalgutkar et al., 2005), which increases the risk of EC with long-term use. Moreover, the conjugated acrylic acid side chains can produce active metabolites leading to adverse reactions in patients.
2) The derivative of AZD9496 (and D24) was substituted with benzoic acid and found to have better drug-forming properties than acrylic acid. SAR analysis indicated that D24 not only had increased binding affinity but also improved degradation potency.
3) Fluorine substituted side chains are also commonly employed in tail side chains, such as in fluoroalkyl azetidine structures, including fluor propyl pyrrolidinyl side chains (SAR439859), fluoroethyl azetidine (GNE-502), fluor propyl azetidine (GNE-149, GDC-9545, AZD-9833), and fluorophenyl sulfonyl (Fulvestrant, ZB-716). The fluorine atoms in these side chains have anti-proliferative activity and are vital for degradation efficiency.
4) Fluorine substitution is also commonly employed in tail side chains, such as in fluoroalkyl azetidine structures, including fluor propyl pyrrolidinyl side chains (SAR439859), fluoroethyl azetidine (GNE-502), fluoropropyl azetidine (GNE-149, GDC-9545, AZD-9833), and fluorophenyl sulfonyl (Fulvestrant, ZB-716). The fluorine atoms in these side chains have anti-proliferative activity and are vital for degradation efficiency.

#### 4.10.1 Backbone

The backbone structure of oral SERDs is typically influenced by older-generation drugs, particularly the SERM parent nucleus. Several novel derivatives of core composition are now available, and natural product structures may provide a new avenue for future SERD core optimization (Figure 5B).

1) MHO7: This ophiobolin derivative was isolated from a mangrove fungal product in a previous study and exhibits high anti-proliferative activity against breast cancer cells, particularly ER+ breast cancer cells. It blocks estrogen by down-regulating ERα at both the mRNA and protein levels (Zhao et al., 2019).
2) XH04: Designed and synthesized by the Lu team, XH04 contains a 7-hydroxy-2H-chromene-3-carbonyl backbone using coumarin as a precursor. It exhibits good degradation potency (IC_50_ = 0.8 μM) and excellent ERα binding affinity in an MCF-7 cell culture model and shows a good pharmacokinetic profile in vitro assays (Lu et al., 2021).
3) XHA1614: This SERD series comprises compounds with a 1,3,5-triazine ring introduced into a highly linked benzyl sequence derived from ER ligands. After screening for antiproliferative activity, the superiorly active XHA1614 was identified. This backbone also provides superior antagonistic activity by inhibiting progesterone receptor mRNA expression in MCF-7 cells (Lu et al., 2020).

## 5 SERCAs

Resistance to targeted ER therapies can arise through various mechanisms, including but not limited to alterations in the expression of related transcription factors, co-regulatory proteins, and cell cycle proteins. Resistance to Tamoxifen treatment is generally associated with the hotspot mutations in Y537 and D538G. These resistance mutations may lead to reduced effectiveness of degraders and antagonists, highlighting the urgent need for the development of novel ERα antagonists. In response to this challenge, scientists have proposed a new approach called targeted covalent inhibitors (TCIs), which holds promise as a strategy for future development (Nettles et al., 2008).

TCIs are a new class of covalent drugs that have been discovered using rational drug design. They offer higher potency, selectivity, and better efficacy. The design of TCIs takes advantage of the formation of covalent bonds between electrophilic reagents on the ligand and nucleophilic centers in the protein after reversible binding. Depending on the traditional drug mechanism of reversible binding or non-covalent interactions between the ligand and its biological target, this specific design results in a significant increase in target binding potency and inhibitory capacity.

In 2018, SERCA was introduced as a novel ERα inhibitor based on the concept of TCIs. SERCA represents an innovative approach in the development of ERα inhibitors. SERCA folds ERα (wild-type and mutant, e.g., Y537S, D538G) into a unique antagonist conformation by targeting a unique cysteine residue (C530). Previous reports have shown that four cysteine residues (381, 417, 447, and 530) in the ER-LBD are covalent targets of ERα, with C530 frequently used to design constructs for covalent antagonists (Harlow et al., 1989).

### 5.1 H3B-5942

Puyang et al. (2018) designed the first SERCA based on the structure of 4OHT: H3B-5942 (**37**). In a previous study, it was demonstrated that mutations in the ERα protein can activate its function, leading to partial resistance to anti-estrogen therapy. Specifically, the Y537 and D538 mutations are located in the AF-2 helix of ERα, which can introduce stable interactions that shift towards the agonist conformation in the absence of ligands, thereby enhancing its activity. To counteract this effect, a new compound called SERCA has been designed to shift the dynamic equilibrium towards an unstable antagonist conformation. This is achieved by targeting the non-conserved cysteine at position 530 of the H11 terminus of the helix, which is present in the ligand binding pocket of ERα. To develop SERCAs, various analogs with different side chains and electrophilic reagents were explored, resulting in the design of a flexible side chain with an internal acrylamide substitution of **10** (Furman et al., 2019). The crystal structure analysis confirmed that the receptor shifts to the desired antagonist conformation in the mutant structure. Specifically, the indazole parent nucleus is anchored to the bottom of the pocket by hydrogen bonding to E353, while the flexible junction extends upwards from the parent nucleus to C530, with which it is covalently bound.

To investigate how the mechanism of **37** and its differences from SERD and SERM, the parental MCF-7 line expressing ERα ^WT^ and the ST941 patient-derived xenograft (PDX) cell line expressing ERα ^Y537S/WT^ were treated with 4OHT, Fulvestrant, and **37** for 24 h. The results showed that **37** and 4-OHT treatment did not alter the ERα protein levels, indicating that the protein was not degraded but instead was conformationally altered. Additionally, the study utilized a mammalian 2-hybrid-based reporter assay with a panel of nuclear receptor coactivators to examine the interaction of a set of coactivators with ERα. It was found that the **37** bounds to ERα ^Y537S^ and ERα ^D538G^ failed to interact with conformation-sensitive peptides that are specifically recruited by SERM- or SERD-bound mutant ERα. This further confirms that **37** induces unique conformational changes that represent a novel antagonistic mechanism for both wild-type and mutant ERα. Unlike SERDs, H3B-5942 binds irreversibly due to the covalent nature of its interaction, allowing **37** to remain for a prolonged period while acting against the antagonistic activity of wild-type and mutant ERα.

### 5.2 H3B-9224

The unsaturated bond of the electrophilic side chain was added to **37**, therefore yielding H3B-9224 (**38**). Proliferation assays showed that a C530 S point mutation in ERα^WT^ reduced the activity of **37**, but had no effect on E2-mediated ER function, and the activity of **38** was not reduced. In assays to determine the antitumor activity of **37** in breast cancer models, significant anti-proliferative activity was demonstrated in both *in vitro* assays and *in vivo* analyses, and the well-tolerated doses of **37** showed significant antitumor activity in both ERα^WT^ and ERα^MUT^ settings. The current ideal dosing pattern involves combining **37** with CDK4/6 or mTOR inhibitors to produce greater inhibitory potency.

The potency of H3B-5942 (**37**) heavily depends on covalent binding, as evidenced by the comparison with **38** which suggests the possibility of an escape mechanism when C530 is mutated (Reese et al., 1991). Therefore, Feynman et al. optimized the parent nucleus scaffold of **37** to obtain H3B-6545 (**39**), which further enhances the antagonistic activity (Furman et al., 2022).

**FIGURE 6:**
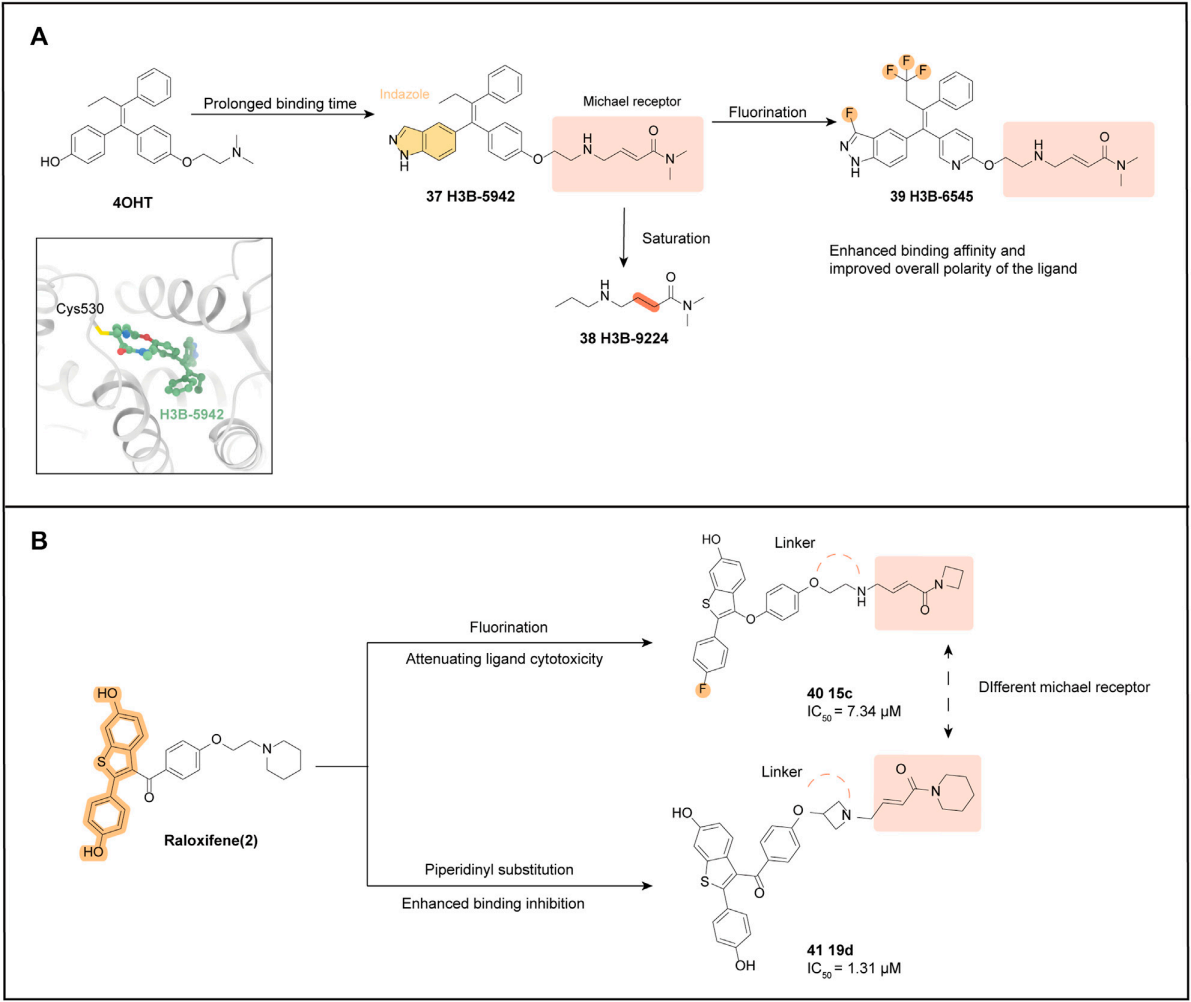
SERCA optimization process. **(A)** optimization processes of H3B-6545 and H3B-9224. The crystal structure of ER with H3B-5942 (Yu et al., 2022) is shown (PDB code: 6CHW); **(B)** optimization processes of two SERCAs based on the structure of Raloxifene.

After observing the crystal structure of ERα ^Y537S^ bound to **37**, the researchers attempted to modify the core with small lipophilic substituents to enhance hydrophobic interactions at the core in the hydrophobic pocket. Hydrogen bonding interactions between the side chain of E353 and the hydrogen of N1 of indazole, and the ability of the pyridine substituent on the C ring to form a hydrogen bond with the T347 residue, not only further enhance the binding affinity but also improve the ligand’s overall polarity. The final choice of the trifluoro substitution of the ethyl side chain, the 3-fluoro substitution of the indazole, and the 3-pyridine substituent at the C ring resulted in a product **39** with the best cellular potency while maintaining acceptable physicochemical properties (Figure 6A).

A significant interaction between the **39** substituted fluorine atoms and H5 and H7, resulted in a conformational change in ERα. Specifically, two residues on H11, H524, and M528, reorient their side chains, while the length of the loop connecting H6 and H7 near H3H3 extends a helical turn at its C-terminus (S338-S341), bringing H3 into direct contact with H5. All these modifications make the ERα ligand binding pocket more compact, which provides for a stronger interaction with the **39** core. Additionally, the results of the jump dilution experiments suggest that the modification of **39**, relative to **37**, makes it less dependent on covalent engagement, which could prolong ERα occupancy and improve antagonistic viability. After demonstrating the antitumor activity of **39** in multiple ERα^WT^ CDX/PDX models, evaluation of three PDX models (ST941 model, ST2177 model, and ST2177 PDX model) showed that **39** demonstrated superiority over Tamoxifen and Fulvestrant in both pharmacodynamic modulation and levels of antitumor activity.

In conclusion, *in vivo* analysis confirmed that H3B-6545 (**39**) has potent single-agent antitumor activity in both ERα^WT^ and ERα^MUT^ settings at well-tolerated doses. Moreover, H3B-6545 showed dose-dependent pharmacokinetics, with 50% oral bioavailability (Ge et al., 2019). Due to its superior preclinical trial performance, the next step of validation and development was undertaken, and clinical trials are currently underway for the treatment of endocrine therapy-resistant ERα+ breast cancer carrying wild-type or mutant *ESR1*. H3B-6545 is being evaluated in clinical phase II (NCT03250676).

### 5.3 Related derivatives of benzothiophene stents

SERCAs can also be designed and optimized based on the benzothiophene backbone scaffold of Raloxifene, resulting in two representative derivatives: **15c** (**40**) and **19d** (**41**). Substituting the 4′position with a fluorine atom attenuates the cytotoxicity of the ligand while maintaining its binding affinity for ERα (Liu et al., 2005). The optimization of H3B-6545 (**39**) combined the idea of introducing an amine electrophilic structure (Michael receptor), similar to H3B-6545, to interact with the C530 site. Additionally, ethyl and azetidine were separately substituted for the linker portion of the molecule obtaining the ethyl linker **40** (Figure 6B) (Bai et al., 2021a).

*In vitro*, tests showed that **40** exhibited significantly higher antiproliferative activity against MCF-7 than Raloxifene (IC_50_ = 7.34 μM VS. 6.56 μM). Furthermore, derivatives with azetidine substitution at the junction harmed antiproliferative potency, suggesting that the ethane junction favors anti-tumor activity. This is likely due to the ethane junction bringing the electrophilic portion closer to C530, as supported by the results of molecular conformational analysis. A further assessment was the determination of binding affinity using a fluorescence polarization scheme, and **40** exhibited excellent binding inhibition. *In vitro* ADME characterization indicated that **40** has good vitro pharmacodynamic parameters.

The same core was used for **40** and **41**, with the Michael receptor replaced by a terminal piperidine group in the hope of enhancing the covalent binding of ER (Bai et al., 2021b). The inhibitory potency of ethyl, piperazinyl, and azetidine linkages was compared at this linkage, and the more potent azetidine-binding compound **41** was ultimately selected, which also showed the strongest binding inhibition in the fluorescence polarization scheme (IC_50_ = 1.31 μM).

Molecular conformation showed that the LBD binding of the **41** core portion and ERα is consistent with normal benzothiophene, but the team also observed irreversible covalent splicing of the electrophilic reagent portion targeting Cys530 (Xiong et al., 2016).

The benzothiophene core is anchored to the bottom of the pocket by hydrogen bonding, and the Michael receptor extends upward to residue C530. Like **37**, **41** can act as a covalent antagonist targeting ERα. Although **41** showed potent antiproliferative activity, the expression of GREB1 and TFF1 was significantly downregulated. Further exploration with additional research and experimental data is still needed to confirm its potency and its potential as a new generation of SERCAs.

## 6 Summary

### 6.1 Outlook

ER has received considerable attention due to its extensive signal transduction pathways, complex crosstalk connections, and its role in various diseases. Breast cancer, which is closely related to ER, has always been a top concern in female malignant tumors (Waks and Winer, 2019). However, the existing treatment options can lead to adverse reactions such as radiodermatitis, skin necrosis, and radiation pneumonia. Additionally, some patients may experience bone marrow suppression after undergoing prolonged radiation therapy. Currently, endocrine therapy is a commonly used treatment approach for patients with multiple hormone-related diseases (Reinbolt et al., 2015). However, endocrine therapy still faces several limitations and one of them is the acquired drug resistance after treatment. It poses a challenge to the long-term effectiveness of endocrine therapy. Therefore, the research and development of a new generation of small molecule antagonists and degradants targeting ER are urgently needed. This article mainly reviews the development of SERDs and SERCAs in recent years, especially regarding the optimal approach, screening methods, and the latest research progress of drugs entering clinical practice.

For now, there are several commonly used methods for screening small molecule drugs, including (Siegel et al., 2020) hybridizing known basic structures to obtain a new ideal chemical scaffold with strong degradation efficiency (Szymiczek et al., 2021), MTS and high-throughput screening (HTS) which can quickly screen the biological target activity of large chemical libraries using automation, miniaturization determination, and large-scale data analysis. For example, hierarchical virtual screening can be performed (Mayr and Bojanic, 2009; Kumar and Zhang, 2015; Zhang et al., 2021a) discovering new parent nuclei based on the chemical structure of natural products, such as XH04 and MH07 obtained from coumarin, which generally possess good pharmacokinetic characteristics (Harbeck et al., 2019), computer-aided drug design using the current calculation level and simulating the interaction between the receptor and the ligand using computer modules to screen for potential drugs. For example, Zhang et al. used the Desmond module in Schrodinger Maestro 2019 to analyze the structure-activity relationship and key amino acids in the protein, and (Jamshidi et al., 2018) chemical modification of the structure of small molecule drugs. The chemical optimization ideas of SERDs are summarized above, including the selection of functional groups, the selection of electrophilic side chains to reduce lipophilicity, fluorine-containing or boric acid-containing side chains, and the selection of side chains such as benzoic acid. With these excellent screening methods, more than ten SERDs with improved drug properties have entered clinical trials over the past decade (Hernando et al., 2021; Bhatia and Thareja, 2023).

In addition, the increased use of SERDs as combination therapy with other inhibitors targeting multiple pathways involved in estrogen signaling and cell cycle progression has brought new hopes for patients with breast cancer and other ER-related diseases (La et al., 2016; Gombos et al., 2023). For example, AZD9496, GDC-9545, and LSZ-102 were each combined with CDK4/6 inhibitors, PI3K-specific inhibitors, and an aromatase inhibitor, respectively (Wardell et al., 2015; Weir et al., 2016; Jhaveri et al., 2021; Liang et al., 2021). The synergistic effects of targeting multiple pathways involved in estrogen signaling and cell cycle regulation have the potential to overcome resistance mechanisms, enhance treatment responses, and ultimately improve outcomes for patients.

In the process of collating and analyzing existing drugs, researchers can continuously discovery and develop new analytical metrics to optimize lead compounds. One such metric is ligand efficiency, which has become an important parameter in lead compound optimization and can enhance the success rate of synthesizing effective lead compounds (Shiao et al., 2013; Reynolds, 2015). In the drugs mentioned in the review, lipophilic ligand efficiency is indeed emphasized. This is because lipophilicity plays a crucial role in the oral bioavailability and therapeutic efficacy of small molecule drugs in the body. Additionally, the ability of a drug to bind to hemoglobin is an emerging area of interest in drug discovery. Exploring the relationship between lipophilic ligand efficiency and the drug’s interaction with hemoglobin could provide valuable insights for optimizing drug design.

### 6.2 Limitations and future directions

However, there are still two major limitations.

The first limitation is resistance to endocrine therapy. Several regulatory mechanisms related to ER can drive the development of resistance against anticancer drugs. Most *ESR1* mutations predominantly occur in the LBD region of the ER. These mutations result in a conformational change of ER, leading to the activation of ER activity even in the absence of ligand binding, including estrogen, SERM, and SERD (Brett et al., 2021). Most drug-resistant patients belong to the *ESR1* mutation, which is relatively mature and controllable (Brett et al., 2021). Current clinical trials have demonstrated that H3B-6545 (**32**) is an effective development option for endocrine resistance: *ESR1*-MUT (NCT03250676). However, some patients may develop a PIK3CA mutation. While endocrine therapy reduces mortality by up to 40% in early-stage disease and is highly effective in controlling metastatic disease, therapeutic resistance remains a momentous clinical issue (Nagy and Jeselsohn, 2022).

#### 6.2.1 Proteolysis-targeted chimerism (PROTAC)

Since ERα can activate transcription without ligands, i.e., the existence of “ligand-independent activation,” the removal of ERα protein by using degradants may become the most promising idea to overcome drug resistance (Osborne and Schiff, 2011; Andreano et al., 2020). Recently, PROTACs have been studied as a potential new protein degradation strategy. PROTACs are bifunctional hybrids that simultaneously bind to a protein of interest (POI) and an E3 ligase to form a ternary complex, inducing E3 ligase to ubiquitinate the target protein and start the subsequent degradation process (Lai and Crews, 2017; Lin et al., 2020; Qi et al., 2021). Moreover, unlike most other small-molecule drug interventions, PROTAC’s action mechanism is “event-driven” rather than competition-driven, allowing smaller doses and relatively smaller drug effects (Salami and Crews, 2017; Burslem et al., 2018; Churcher, 2018). Compared with other small molecule drugs, the greatest advantage of PROTAC technology is its huge potential to target many non-pharmaceutical proteins. ARV-471 is the first oral organism to enter the clinic, which can utilize ER PROTAC to conduct biomarker analysis. The results show that ER undergoes strong degradation (∼200%) regardless of the existence of *ESR1* mutation. In 2023, Xie et al. disclosed a series of degradants based on the oxabicycloheptane sulfonamide (OBHSA) scaffold, among which compound ZD12 exhibited excellent anti-tumor efficacy and ERα degradation activity superior to Fluvastatin in both Tamoxifen-sensitive and -resistant breast cancer mouse models (Burslem and Crews, 2020). However, there are still many challenges to be addressed, such as the low oral bioavailability of PROTACs, the occurrence of off-target effects, and the limited development of E3 ubiquitin ligases (Garber, 2022; Xie et al., 2023).

#### 6.2.2 The cancer antibody-drug coupling (ADC)

An immune conjugate consisting of a monoclonal antibody and a cytotoxic drug that enables the selective delivery of the drug to the target cancer cells. And the impact of drug resistance gives good prospects for the development of this novel molecule (Chau et al., 2019).

#### 6.2.3 Delve into the ER signaling pathway

This year Lu’s team modified G1T48, a clinical candidate SERD, by attaching it to the NHC-gold (I) complex as a dual-targeted drug targeting both the ER and an oxidoreductase, TrxR, and the experiments have shown that several of the compounds are highly effective. This study provides new insights and directions for future development strategies for SERDs, bringing investigators’ attention to dual-targeted drug development and the study of the ER signaling pathway (Lu et al., 2023).

The second limitation is the side effects of SERDs on the uterus. Some oral SERDs have shown partial agonistic activity in the uterus, leading to an increase in uterine wet weight during preclinical studies with mice (Patel and Bihani, 2018). This poses concerns regarding the safety of SERDs for use in breast cancer treatment, as it may promote uterine hyperplasia or other adverse effects.

The development of complete estrogen receptor antagonists (CERAN) represents a relatively new approach to enhancing therapeutic performance. CERANs aim to achieve complete and consistent antagonism of the ER. By completely blocking the transcription activation domain in the ER, CERANs prevent the receptor from activating target genes and exerting its oncogenic effects. One notable CERAN, named OP-1250, is currently undergoing clinical study and showing promising results in pharmacodynamics and anti-tumor activity (NCT04505826) (Hamilton et al., 2022). The development and evaluation of CERANs hold great potential for enhancing the targeted treatment of ER-positive cancers. Further research and clinical trials will provide further insights into their effectiveness and safety.

In conclusion, given the importance of inhibiting ER activity and signaling in the treatments of breast cancer and other ER-related diseases, developing more potent and selective inhibitors of ER to overcome resistance and improve treatment outcomes is still of great importance. Additionally, advancements in biotechnology and chemical screening techniques have facilitated the discovery and optimization of new compounds that can act as ER degraders or antagonists. Moreover, the optimization direction of SERDs and SERCAs is now expanding and becoming more diversified. New research and development efforts are exploring the potential of dual-targeted drugs that target ER and oxidoreductase enzymes simultaneously and combination therapies to regulate other alternative receptors or proteins implicated and ER as well (Lu et al., 2023). By targeting multiple pathways or receptors simultaneously, the aim is to enhance the effectiveness and overcome potential resistance mechanisms of SERDs and SERCAs. This approach may offer new opportunities in the development of novel SERD and SERCAs -based therapies.
